# Medical errors during training: how do residents cope?: a descriptive study

**DOI:** 10.1186/s12909-021-02850-1

**Published:** 2021-07-29

**Authors:** Saba Fatima, Stefania Soria, Nora Esteban- Cruciani

**Affiliations:** 1 Division of Hospital Medicine, Department of Pediatrics, University of Kansas School Of Medicine- Wichita, 3243 E Murdock, Suite 402, Wichita, Kansas 67208 USA; 2 Department of Pediatric Cardiology, Rush University Medical Center, Chicago, USA; 3 Department of Pediatrics and Adolescent Medicine, Einstein Medical Center, Philadelphia, USA

**Keywords:** Medical errors, Patient safety, Burnout, Resident wellness, Coping strategies, Depression

## Abstract

**Background:**

Physicians’ self-perceived medical errors lead to substantial emotional distress, which has been termed the “second victim phenomenon.” Medical errors during residency are associated with increased burnout and depression. It is important to know how residents cope with self-perceived medical errors and how they gain personal and emotional support in order to develop effective interventions.

**Objective:**

To assess the impact of self-perceived medical errors on residents’ well-being, the range of coping strategies during training, and the extent of personal and institutional support.

**Methods:**

An online cross-sectional survey was administered via email in October 2018 to 286 residents across all specialties in a 548-bed single urban academic medical center. The survey covered three domains focusing on residents’ most serious self-perceived medical error: (1) emotional response, (2) coping strategies using the BRIEF COPE Inventory, and (3) personal and institutional support.

**Results:**

109/286 residents from various specialties responded. Internal Medicine, Pediatrics and Emergency Medicine constituting 80 % of respondents. Self-perceived medical errors during residency were widespread (95 %). One in five medical errors was classified as moderate to severe. Most residents acknowledged a sense of guilt, remorse and/or inadequacy. Use of maladaptive coping strategies was high. Open-ended responses pointed to fear of retaliation, judgement, shame and retribution. Most residents disclosed their error to a senior resident but did not discuss it with the patient’s family. Only 32 % of residents participated in a debriefing session.

**Conclusions:**

Most residents were directly involved in medical errors, which affected their emotional well-being. The use of maladaptive coping strategies was high. Residents’ fear of consequences prevented disclosure and discussion of self-perceived medical errors. This information is relevant to implement targeted interventions.

**Supplementary Information:**

The online version contains supplementary material available at 10.1186/s12909-021-02850-1.

## Background

Medical errors not only have an impact on patient safety but also have an emotional bearing on the medical providers caring for affected patients. Following a medical error, most medical providers experience a broad range of feelings including guilt, remorse, anger and inadequacy [1, 2]. The suffering of medical providers in the face of a serious medical error has become known as the second victim phenomenon, which may result in a period of profound professional and personal anguish, described by Wu in 2001 [3]. *“Second victims are health care providers who are involved in an unanticipated adverse patient event, in a medical error and/or a patient related injury and become victimized in the sense that the provider is traumatized by the event. Frequently these individuals feel personally responsible for the patient outcome. Many feel as though they have failed the patient, second guessing their clinical skills and knowledge base” *[4]. As frontline providers, residents are susceptible to the second victim phenomenon. In a study involving Internal Medicine residents, West et al. reported significant distress after a self-perceived medical error [5]. Other studies on medical errors by residents have revealed an association between self-perceived medical errors and decreased quality of life, burnout, fatigue, depression, and loss of empathy [5, 6].

The range of emotional responses after experiencing a distressful event typically involves adaptive and maladaptive coping strategies. Adaptive coping strategies include acceptance, planning, humor, active coping, positive reframing, use of institutional and emotional support. Maladaptive coping strategies include denial, venting, self-blame, self-distraction, substance abuse, and behavioral disengagement [7]. Only a few studies have explored the variety of coping mechanisms used by residents after a self-perceived medical error. Wu et al. and Engal et al. described adaptive coping strategies, such as discussion of a medical error with colleagues, as central to emotional recovery among residents [8, 9]. In contrast, Meyer et al. and Alveal et al. described maladaptive coping strategies to be associated with depression and perceived stress in non-medical fields [10, 11]. Little is known about residents’ maladaptive coping strategies and emotional support sought after a medical error. Understanding the range of coping strategies that residents use after a medical error may help us to better appreciate and provide the support they need to foster their emotional recovery and to minimize burnout and depression.

Our study aims to explore the emotional impact of medical errors, the range of adaptive and maladaptive responses, and the use of institutional support systems by physician residents in various residency training programs.

## Methods

We conducted an online cross-sectional survey of residents at an urban academic medical center. The medical center is a 548-bed teaching hospital, with 286 residents in nine residency programs, including Internal Medicine (*n* = 89 residents), Emergency Medicine (*n* = 58), Psychiatry (*n* = 32), Pediatrics (*n* = 31), General Surgery (*n* = 21), Obstetrics and Gynecology (*n* = 19), Urology (*n* = 12), Radiology (*n* = 12), Neurology (*n* = 12)and Orthopedics (*n* = 12). At the time of the study, there was no known program to address second victim phenomenon at this institution. In October 2018, we e-mailed an electronic survey to all residents at our medical center. Respondents could answer anonymously. A reminder survey was e-mailed two weeks after the initial request. Chief residents for each of the residency programs were sent separate emails for assistance. Residents were reminded during educational conferences to fill out the survey. The study was deemed as exempt by the medical center’s Institutional Review Board.

### Survey instrument

The survey instrument was constructed after a thorough review of the literature and was refined after a one-month pilot period. The survey covered three domains focusing on “the most serious medical error” that residents recalled having been involved during residency.

First, we asked residents if they had ever been directly involved in a medical error, and if they had been, they were prompted to focus on their “most serious medical error” in order to explore a single event, likely associated with the resident’s most profound emotional burden. We used the World Health Organization’s international classification for patient safety reporting to define the potential harm severity of a resident’s self-perceived “most serious” medical error as: near miss, minimal, moderate, severe, and death [12].

The survey domains included:

1. Emotional response: We asked residents to identify the range of emotional responses following their most serious medical error while in training, including guilt, remorse, anger, inadequacy, indifference, and no particular feeling. We requested them to grade the degree of each of these emotional responses on a five-point Likert scale format (1, 2 = not at all to a small extent, and 4, 5 = moderate to a great extent).
2. Coping Strategies: Coping strategies were assessed using the BRIEF COPE Inventory, a validated abbreviated version of the Coping Orientation to Problems Experienced Inventory (COPE, Carver et al. [7]). The BRIEF COPE Inventory is a multidimensional measure of strategies used to assess a broad range of coping responses to stressors. It includes 28 items assessing 14 coping strategies: self-distraction, active coping, denial, substance use, use of emotional support, use of institutional support, behavioral disengagement, venting, positive reframing, planning, humor, acceptance, religion, and self-blame. Respondents rate items on a 4 four-point Likert scale, ranging from 1 (“I haven’t been doing this at all”) to 4 (“I’ve been doing this a lot.”). Each coping strategy comprises two paired items in the survey that were ranked individually, thus the total score for each category ranges from 2 (minimum) to 8 (maximum). As an example, “self- blame” on the questionnaire comprises “I have been criticizing myself” and “I have been blaming myself for things that happened”. Higher scores indicate increased utilization of that specific coping strategy. Based on prior studies, we separated the Brief COPE Inventory responses into maladaptive vs. adaptive coping strategies [10, 11].
3. Personal and Institutional support: This domain focused on residents’ personal and institutional support after their most serious medical error. This section included questions regarding the residents’ ability to discuss their medical error and to receive support from colleagues and/or faculty members, using a five-point Likert scale (1, 2 = not at all or little to 4, 5 = great deal or a lot). In addition, we asked residents if they had the opportunity to participate in a debriefing session after the event. Lastly, we included an open-ended question to inquire about any self-perceived barriers that may have precluded disclosure and discussion of medical errors. A Word-Cloud Generator was used to analyze the most common word responses identified in the open-ended section.

Data were analyzed with SPSS version 20.0. We summarized respondents’ demographics using standard descriptive statistics. Categorical variables were expressed in frequencies and percentages; mean and standard deviation were reported for continuous variables.

## Results

A total of 109/286 (38 %) residents responded to the survey. Internal Medicine (32 %), Pediatrics (28 %) and Emergency Medicine (20 %) constituted 80 % of respondents (Table 1). Response rate varied by specialty: Pediatrics (100 %), Internal Medicine (39 %), Emergency Medicine (38 %), Neurology (38 %), Obstetrics and Gynecology (37 %), General Surgery (24 %), Surgical Subspecialties (14 %), and Psychiatry (9 %). All levels of training (PGY1-PGY6) were represented, as well as both international and American medical graduates (Table 1).

**Table 1:** Demographics of responding residents (*n* = 109)

		n (%)
Post-graduate Year (PGY) Level	PGY 1	34 (32)
	PGY 2	32 (29)
	PGY 3	31 (28)
	PGY 4–6	12 (11)
Gender	Male	45 (41)
	Female	64 (59)
Specialty	Internal Medicine	35 (32)
	Pediatrics	31 (28)
	Emergency Medicine	22 (20)
	Obstetrics and Gynecology	7 (6)
	General Surgery	5 (5)
	Psychiatry	3 (3)
	Surgical Subspecialties	3 (3)
	Neurology	3 (3)
Medical School	American	41 (38)
	Foreign	68 (62)

### Residents’ self-perceived involvement in medical errors

Almost all residents (95 %) reported being directly involved in at least one self-perceived medical error (Table 2). Some residents reported multiple events. One in five medical errors was classified as moderate to severe (life-threatening). Respondents indicated that 7 % of errors might have resulted in permanent consequences to the affected patients. Most errors occurred during PGY1 level of training (58 %), compared to PGY2 (25 %) and PGY 3 (12 %).

**Table 2:** Involvement in self-perceived medical errors during residency

Type of Error	% of residents (n)
None	5 (5)
NEAR-MISS (an error occurred but did not reach the patient)	76 (82)
MINIMAL (an error reached the patient, causing minimal or no detectable harm)	45 (49)
MODERATE (moderate patient harm, requiring intervention)	13(14)
SEVERE (life-threatening, requiring intervention to sustain life	6(6)
Death	1(1)

**Fig. 1:**
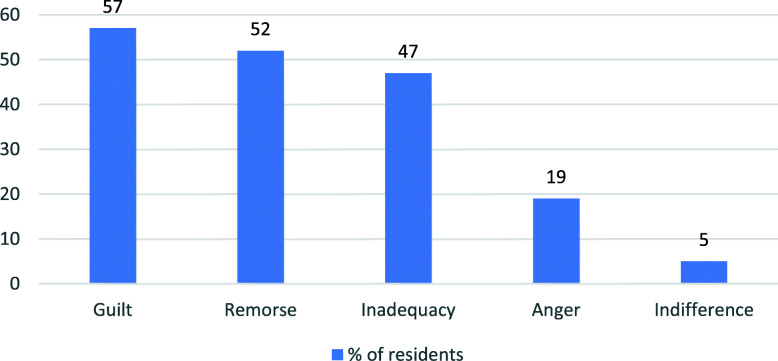
Residents emotional response to their most serious medical error

Responses to the three survey domains are categorized below:

1. Residents’ emotional response to their most serious medical error: The majority of residents acknowledged guilt (57 %) or remorse (52 %) to a moderate or great extent after the safety event. Almost half (47 %) experienced inadequacy, while 19 % experienced anger and 5 % reported indifference (Fig. 1).

2) Residents’ coping strategies after a self-perceived serious medical error: The BRIEF COPE Inventory responses indicated that residents used the following coping strategies in order of frequency: planning, active coping, self-blame, use of institutional support, use of emotional support, acceptance, self-distraction, and positive reframing. Strategies used less frequently by trainees were venting, humor, religion, behavioral disengagement, substance use and denial (Table 3).

**Table 3:** Residents’ Coping Strategies for Self-Perceived “Most Serious” Medical Errors

Coping Strategy^a^	Mean	SD
***Adaptive***		
Planning	5.56	± 0.98
Active coping	5.52	± 0.98
Use of institutional support	5.12	± 0.95
Use of emotional support	4.90	± 0.96
Acceptance	4.68	± 0.93
Positive reframing	4.08	± 0.93
Humor	3.16	± 0.92
Religion	3.00	± 0.86
***Maladaptive***		
Self-blame	5.16	± 0.96
Self-distraction	4.64	± 0.96
Venting	3.82	± 0.84
Behavioral disengagement	2.80	± 0.69
Substance abuse	2.54	± 0.61
Denial	2.36	± 0.50

^a^Each coping strategy comprises of two questions on the survey, ranked individually on a 4- point Likert scale. Minimum score = 2, maximum score = 8

3) Residents’ peer and institutional support after a serious medical error: Most residents disclosed their most serious self-perceived medical error to a resident above their level of training, less than half discussed the event with an attending, and only one in four talked to the patient or family involved in the event (Table 4).The majority of residents felt supported by their colleagues, however one in four residents did not feel supported by the faculty (Table 5). Only one-third (32 %) residents participated in a debriefing session after the medical error was disclosed. Half of the residents did not know if debriefing sessions were held in their departments.

**Table 4:** Disclosure after a Serious Medical Error during Residency

		% of Residents (n)
Disclosure and discussion	Resident above level	62 (60)
	Resident at or below level	59 (57)
	Attending physician	47 (46)
	Friends or family	36 (35)
	Nurse	26 (25)
	Patient	25 (24)
	Program director	11 (11)
	Did not discuss	8 (8)

**Table 5:** Support after a Serious Medical Error during Residency

		% of Residents (*n* = 97)
Support by peers	A great deal or a lot	55 (53)
	Moderate	26 (25)
	Little or not at all	19 (19)
		% of Residents (*n* = 96)
Support by faculty	A great deal or a lot	44 (42)
	Moderate	22 (21)
	Little or not at all	25 (24)
	Not applicable	9 (9)

Sixty-four residents (59 %) provided open-ended responses regarding factors that might have precluded them from disclosing medical errors to a supervisor within their training program. Most common themes amongst the responses were fear (25 %), judgement and loss of image (20 %), retribution and consequences (15 %), shame and guilt (8 %). The various fears noted by the residents included fear of retaliation and being reprimanded, fear of loss of trust, fear of being labeled as incompetent or negligent and fear of consequences. Other response themes included concern about high or unrealistic expectations, perfectionism and fear of being labeled weak or inadequate if they disclosed a mistake. Some residents pointed to a prior negative experience with a colleague and lack of kindness in previous milder situations as a reason for non-disclosure. Some quotes from residents included: “I could not admit that I had failed or made a mistake”, “I felt like I was always being judged”, “I shared it once and was told, well you need to be more careful”, “I did not want to look like a weak resident”, and “doctors are not allowed to make mistakes”.

## Discussion

Our study highlights that self-perceived medical errors by residents are associated with profound emotional responses, leading to a range of adaptive and maladaptive coping strategies, with inconsistent institutional support. The most common emotional responses were fear, shame and feeling judged. Most residents in this study reported using adaptive coping strategies after a medical error, such as planning, active coping, emotional and institutional support. However, many residents reported also using maladaptive coping strategies including self-blame and self-distraction, followed by venting, substance abuse, behavioral disengagement, and denial. Two prior studies that explored residents’ coping strategies after facing medical errors found that most residents cope by discussion of their error [8, 9]. To our knowledge, this is the first study using a validated coping scale to explore the range of adaptive and maladaptive coping strategies used by residents after a medical error across multiple specialties. This gives current graduate medical education leaders objective insight into resident behaviors, providing opportunities to offer support and improve resident burnout.

In our study, residents revealed fear of retaliation, retribution, and judgment after a medical error, which likely reflects the culture of perfectionism in medicine [2]. Most residents expressed that fear of consequences after an event prevented them from disclosing and discussing their error with their supervisory faculty and the patient or family involved in the event. Christensen et al. conducted in-depth interviews with physicians on the impact of making clinical mistakes. They found that perfectionism and competitiveness engrained in medical school were the main factors causing distress to clinicians about medical errors [2]. Abraham Verghese has described the medical professionals’ attitude as “a silent but terrible collusion to cover up pain, to cover up depression; there is a fear of blushing, a machismo that destroys us” [13].

Most residents reported that they did not have the opportunity to participate in a debriefing session after an adverse event and many were unaware if this type of session was available in their residency program or their institution. Most residents reported not feeling supported by faculty members. These findings are important, as prior studies have revealed an association between self-perceived medical errors and decrease in quality of life, burnout, fatigue, depression, and loss of empathy [5, 6]. Fahrenkopf et al. have also described that residents affected by depression made significantly more medical errors than their non-depressed peers, leading to a vicious cycle [14].

Our findings indicate that there is a great a need for early career physicians to receive emotional and educational support to strengthen their ability to develop adaptive coping skills. There is emerging evidence that the use of maladaptive coping strategies by residents who avoid disclosure and discussion, because of fear or other reasons, may adversely affect their personal well-being and lead to depression and burnout.

New strategies to support residents may be needed. These could potentially be achieved by incorporating disclosure, discussion, and responses to medical errors into the standard residency curriculum, by organizing emotional support groups, and by providing skill training and debriefing sessions in response to medical errors. These open discussions could also help residents develop improved emotional intelligence, which has shown to be associated with better coping and less burnout [15]. Faculty development could focus on the increased awareness and skills needed to facilitate supportive discussions of medical errors with trainees.

Our study has several limitations. This is a single-center study, which limits its generalizability. We had a relatively low response rate even after two attempts, which is not uncommon among this type of survey, but may have failed to capture the full range of residents’ perspectives at our institution. Medical errors in our study were self-reported and thus subject to selective recall bias. Some residents who were involved in a medical error may have chosen not to respond, even anonymously. A strength of our study is the inclusion of residents, both male and female, from multiple specialties, PGY levels and educational backgrounds.

Future studies should focus on institutional and educational changes to address the complexity of the second victim phenomenon among residents, and to develop strategies to decrease maladaptive responses to medical errors, including disclosure skills, root-cause analyses, post-event debriefing, blame-free discussions, support groups, faculty development, and institutional resources.

## Supplementary Information

**Additional file 1.**

## Data Availability

The datasets used and/or analyzed during the current study are available from the corresponding author on reasonable request.
