# Supplementary material for: Medical errors during training: how do residents cope?: a descriptive study
Source: BMC Med Educ. 2021 Jul 29;21:408. doi: 10.1186/s12909-021-02850-1 (PMC8320044; doi:10.1186/s12909-021-02850-1)
Supplement: Supplementary file 1 — Additional file 1. [file 12909_2021_2850_MOESM1_ESM.pdf]

MEDICAL ERRORS DURING TRAINING: HOW DO RESIDENTS COPE?: A DESCRIPTIVE STUDY

Authors:

1.Saba Fatima MD

Assistant Professor, Division of Hospital Medicine, Department of Pediatrics, University of Kansas School of Medicine, Wichita, Kansas

2.Stefania Soria MD

Fellow, Department of Pediatric Cardiology, Rush University Medical Center, Chicago

3.Nora Esteban- Cruciani MD, MS

Pediatric Medical Director MBU, Department of Pediatrics and Adolescent Medicine, Einstein Medical Center, Philadelphia

This study was presented orally in Pediatric Academic Societies Meeting 2019

Correspondence to Saba Fatima, Department of Pediatrics, School of Medicine, University of Kansas, Wichita, Kansas, 620 N Carriage Pkwy, Wichita, KS 67208

sfatima@kumc.edu

215-512-3421

Manuscript: 2234 wor**ds**

Medical Errors during Residency

* 1. Are you a resident physician currently completing training in the US?

- Yes
- No

* 2. Please select the option that best describes your current residency?

- Pediatrics
- Internal Medicine
- Emergency Medicine
- Obstetrics/ Gynecology
- Family Medicine
- Psychiatry
- General Surgery
- Surgical Subspecialty
- Neurology
- Radiology
- Dental

Other (please specify)

* 3. What is your current year of training?

- PGY1
- PGY2
- PGY3
- PGY4
- PGY5
- PGY6
- PGY7

Medical Errors

Most physicians are involved in medical errors. We want to understand what coping strategies help trainees encountering medical errors during their residency training. Please, take 7 minutes to candidly answer this anonymous survey and help us with your valued feedback.

* 4. All responses are anonymous. Please, indicate if you have been DIRECTLY INVOLVED in any of the following medical errors during your residency training?
(check ALL that apply)

- [ ] NONE(never involved in a medical error)
- [ ] NEAR-MISS (an error occurred but did not reach the patient) - e.g. dosing error prevented by pharmacy
- [ ] MINIMAL (an error reached the patient, causing minimal or no detectable harm) - e.g. minor diagnostic error
- [ ] MODERATE (moderate patient harm, requiring intervention) - e.g. adverse event from known drug allergy
- [ ] SEVERE (life-threatening, requiring intervention to sustain life) e.g. failure to act on a life-threatening lab result
- [ ] DEATH

* 5. Thinking of your “most serious medical error" , did it had a transient or permanent effect on your patient: (check ONE)

- ( ) TRANSIENT (transient harm, required initial but not prolonged intervention)
- ( ) PERMANENT (permanent harm, required prolonged intervention or hospitalization)

Emotional response to Medical Errors

We are interested in understanding how residents cope with the potential emotional impact that medical errors can have.

* 6. Thinking about your most serious medical error during your residency training, how would you describe your feelings after the event?

	not at all	to a small extent	to some extent	to a moderate extent	to a great extent
Remorse	◯	◯	◯	◯	◯
Guilt	◯	◯	◯	◯	◯
Inadequacy	◯	◯	◯	◯	◯
Anger	◯	◯	◯	◯	◯
Indifference	◯	◯	◯	◯	◯
No particular feeling	◯	◯	◯	◯	◯

Other (please specify)

Communication regarding medical errors

* 7. Thinking about your single most serious medical error during your residency training, who did you discuss it with after the event happened?
check ALL that apply

- [ ] Resident at or below your level of training
- [ ] Resident above your level of training
- [ ] Attending
- [ ] Program Director
- [ ] Nursing
- [ ] Patient or family involved in the event
- [ ] Your own friends or family
- [ ] Did not discuss with anyone
- [ ] Other (please specify)

8. When you think about the time of your single most serious medical error during your residency training, did you feel supported by your COLLEAGUES?

- ○ A great deal
- ○ A lot
- ○ A moderate amount
- ○ A little
- ○ None at all

9. When you think about the time of your single most serious medical error during your residency training, did you feel supported by your FACULTY?

- ○ A great deal
- ○ A lot
- ○ A moderate amount
- ○ A little
- ○ None at all
- ○ Other or Not applicable

10. When thinking about discussing your most serious medical error with supervisory faculty in your training program, what would you say was the most important factor that prevented you from sharing your experience with your supervisors?
Examples include: A prior negative experience with you or a colleague

11. Does your residency program offer debriefing sessions for residents involved in medical errors?

- ◯ Yes
- ◯ No
- ◯ Not sure

Coping Strategies

Different people deal with things in different ways.
The following section focuses on your coping strategies after a medical error. Each item says something about a particular way of coping

12. Thinking about the single most serious medical error you were involved in, mark each item to what extent you did it after the error. Please don't mark on the basis of whether it helped or not.
Rate each item separately in your mind from others.

	Not at all	A little bit	A medium amount	A lot
Turned to work or other activities to take my mind off things	○	○	○	○
Concentrated my efforts on doing something about the situation I am in	○	○	○	○
Saying to myself " This isnt real"	○	○	○	○
Using alcohol or other drugs to make myself feel better	○	○	○	○
Getting emotional support from others	○	○	○	○
Gave up trying to deal with it	○	○	○	○
Taking action to try to make the situation better	○	○	○	○
Refused to believe that it had happened	○	○	○	○
Said things to let my unpleasant feelings escape	○	○	○	○
Got help and advice from other people	○	○	○	○

Coping Strategies

13. Same as before , thinking about the single most serious medical error you were involved in, mark each item to what extent you did it after the error. Please don't mark on the basis of whether it helped or not. Rate each item separately in your mind from others.

	Not at all	A little bit	A medium amount	A lot
Using alcohol or other drug to help me get through it	◯	◯	◯	◯
Tried to see it in a different light, to make it seem more positive	◯	◯	◯	◯
Criticized myself	◯	◯	◯	◯
Tried to come up with a strategy about what to do	◯	◯	◯	◯
Getting comfort and understanding from someone	◯	◯	◯	◯
Giving up the attempt to cope	◯	◯	◯	◯
Looked for something good in what was happening	◯	◯	◯	◯
Made jokes about it	◯	◯	◯	◯
Did something to to think about it less such as going to movies, watching TV, reading, daydreaming or shopping	◯	◯	◯	◯
Expressing my negative feelings	◯	◯	◯	◯
Trying to find comfort in religion or spiritual beliefs	◯	◯	◯	◯
Tried to get advice or help from other people about what to do	◯	◯	◯	◯
Learned to live with it	◯	◯	◯	◯
Thought hard about what steps to take	◯	◯	◯	◯
Blamed myself for things that happened	◯	◯	◯	◯

You re almost done! Just a few demographic questions

14. What WAS YOUR LEVEL of training, when your single most serious medical occurred?

- ○ PGY1
- ○ PGY2
- ○ PGY3
- ○ PGY4
- ○ PGY5
- ○ PGY6
- ○ PGY7

15. Which best describes the population of your CURRENT residency program? (Check ONE)

- ○ Urban , inner city population
- ○ Urban, NOT inner city population
- ○ Suburban
- ○ Rural

16. How many residents in TOTAL are currently training in your program?

- ○ less than or equal to 20
- ○ 21-40
- ○ 41-60
- ○ 61-80
- ○ greater than or equal to 81

17. Did you graduate from?

- ○ Us Medical School
- ○ Foreign Medical School

18. What is your gender?

- ○ Male
- ○ Female

19. With which racial/ethnic group do you identify yourself? Check ALL that apply

- ☐ White
- ☐ Hispanic/Latino
- ☐ Black/ African American
- ☐ Asian
- ☐ Native Hawaiian/other Pacific Islander
- ☐ American Indian/ Alaska Native
- ☐ Other
